# Four New Species of *Nepenthes* L. (*Nepenthaceae*) from the Central Mountains of Mindanao, Philippines

**DOI:** 10.3390/plants3020284

**Published:** 2014-06-06

**Authors:** Thomas Gronemeyer, Fulgent Coritico, Andreas Wistuba, David Marwinski, Tobias Gieray, Marius Micheler, François Sockhom Mey, Victor Amoroso

**Affiliations:** 1 Institute of Molecular Genetics and Cell Biology, Ulm University, 89081 Ulm, Germany; 2 Center for Biodiversity Research, Central Mindanao University, University Town, 8710 Musuan, Bukidnon, Philippines; 3 Friedhofweg 4, 88437 Maselheim, Germany; 4 In der Messe 27, 70327 Stuttgart, Germany; 5 Losburgstrasse 39, 73776 Altbach, Germany; 6 Frankfurter Ring 3, 80807 Munich, Germany; 7 3 rue Frédéric Chopin, 59320 Haubourdin, France

**Keywords:** carnivorous pitcher plants, *Nepenthes*, biodiversity, Philippines

## Abstract

Together with the islands of Sumatra (Indonesia) and Borneo (Indonesia, Malaysia), the Philippines are the main center of diversity for carnivorous pitcher plants of the genus, *Nepenthes* L. *Nepenthes* are the largest of all carnivorous plants, and the species with the biggest pitchers are capable of trapping and digesting small amphibians and even mammals. The central cordillera of Mindanao Island in the south of the Philippines is mostly covered with old, primary forest and is the largest remaining cohesive, untouched area of wilderness in the Philippines. In a recent field exploration of two areas of the central cordillera, namely Mount Sumagaya and a section of the Pantaron range, four new taxa of *Nepenthes* were discovered. These four remarkable new species, *N. pantaronensis*, *N. cornuta*, *N. talaandig* and *N. amabilis*, are described, illustrated and assessed.

## 1. Introduction

The Philippines are nowadays considered to host the highest diversity of carnivorous pitcher plants of the genus, *Nepenthes*. Local conflicts have hindered exploration of the islands in the past, and therefore, only 15 distinct species were known by the late 1990s [1]. In the past decade, a series of field expeditions (mainly to Mindanao and Palawan) has led to the discovery of 11 new taxa ([1] and references therein), while two more species, *N. peltata* Sh.Kurata and *N. robcantleyii* Cheek, were described based on cultivated material [2,3]. More recently, *N. viridis* Micheler from Dinagat Island was described [4]. The description of 11 further species based on herbarium material and the resurrection of *N. graciliflora* Elmer [5,6,7,8,9,10,11] raises the number of Philippine species to 40, more than on the islands of Borneo and Sumatra. However, Jebb and Cheek note that some of their new taxa (e.g., *N. extincta* Cheek) might be “extinct in the wild, due to habitat destruction” [5]. 

The islands of Mindanao and Palawan are the Philippines’ main centers of diversity for *Nepenthes*. Most species occur in the intermediate tropical highland climate prevalent on the numerous mountains of the islands. Diversity hotspots are mountains with ultramafic soils, such as Mt. Hamiguitan in Davao Oriental province (five species in total, three of these endemic) or the Diwata Mountains in Agusan del Norte.

The most extensive mountain massif on Mindanao is the Pantaron mountain range, the major part of the central cordillera of Mindanao. It runs south from the municipality of Claveria in the north of the island towards the south to the municipality of San Fernando and separates the provinces of Bukidnon in the west and Agusan del Sur in the east. The average altitude of this mountain range is approximately 1,000 m, with summits of around 1,500 m that peak out of the cordillera. Almost the whole area is covered by dipterocarp, lower and upper mountain forest and is designated as a key biodiversity area by the Philippine Department of Environment and Natural Resources. The Pantaron range is flanked by the marshes of the Agusan River in the east and by the Pulangi River in the west, the latter being the longest river in Mindanao. Along the Pulangi River, large areas of lower mountain forest have been cleared for agriculture. 

West of the Pulangi River, opposite the Pantaron range, the Tago mountain range marks the western border of the central cordillera. The northern border of the central cordillera consists of a conglomeration of prominent peaks with altitudes significantly above 2,000 m, namely Mt. Sumagaya, Mt. Mangabon and Mt. Kimangkil. While other prominent mountains on Mindanao, like Mt. Apo, Mt. Kitanglad, Mt. Hamiguitan or Mt. Matutum have been explored extensively by local and foreign botanists, the central cordillera has remained unexplored until recently. Large areas of these mountains form part of the ancestral territories of local indigenous tribes, and separatist conflicts between the New People’s Army (NPA) and the military long hindered safe access to this remote area.

A minor peak in the Pantaron range in the San Fernando area was explored by one of the authors in 2008 [12], and Mt. Kiamo in the Tago range was explored in 2011. During the exploration of Mt. Kiamo, the common *N. mindanaoensis* Sh.Kurata, *N. merrilliana* Macfarlane and *N. truncata* Macfarlane were found, as well as two remarkable new taxa that were previously unknown, *N. pulchra* Gronem. and *N. ceciliae* Gronem. [13,14]. *N. pulchra* is an unusually large species that is related to *N. petiolata* Danser, whereas the gracile *N. ceciliae* shares features with *N. copelandii* Merr. ex Macfarlane and was subsequently grouped with the *N. alata* complex of species [5,8]. 

These findings, together with the identification of several species, including *N. surigaoensis* Elmer, on minor peaks in the Pantaron range near San Fernando [12], led to the assumption that the Pantaron range and the adjacent mountains might represent a huge area of diversity for carnivorous pitcher plants. 

In order to verify this theory, an area of the Pantaron range approximately 60 km north of San Fernando and Mt. Sumagaya, one of the high peaks forming the northern border of the central cordillera, were chosen for further field research. This expedition led to the discovery of four additional new *Nepenthes* taxa. The taxonomic descriptions of these new species are provided in this article. 

## 2. Results and Discussion

### 2.1. Taxonomic Description of Nepenthes pantaronensis

*Nepenthes pantaronensis* Gieray, Gronem., Wistuba, Marwinski, Micheler, Coritico, V.B. Amoroso, spec. nov.

#### Diagnosis:

Differs from *N. pulchra* Gronem. in having 2 longitudinal nerves (*N. pulchra*: 3–4), 2 non-fringed or barely fringed wings on the lower pitchers and no wings on the upper pitchers (*N. pulchra*: fringed wings on the lower pitchers and wings reduced to ribs on the upper pitchers) and in having basal stem leaves with a canaliculate petiole (*N. pulchra*: broad-winged petiole). 

#### Type:

Philippines, Mindanao Island, Bukidnon Province, Pantaron mountain range, Mt. Gaka (1,390 m) near Sitio Mahayag (Barangay St. Peter, Malaybalay City, Bukidnon Province, Philippines), 15.08.2012, *T. Gronemeyer and F. Coritico*, holotype CMUH00008625, Central Mindanao University Herbarium (CMUH), Musuan, Bukidnon, Philippines. 

#### Etymology:

The specific epithet denotes that *N. pantaronensis* was discovered in the Pantaron mountain range. 

#### Description:

The *stem* is up to 3 m long, cylindrical in the cross-section, 0.9–1 cm in diameter, with internodes up to 13 cm long.

*Leaves of the basal stem* are linear to oblong, up to 28 cm long and 5 cm wide, with two longitudinal veins on each side of the midrib and numerous pinnate veins running obliquely towards the leaf margins. The apex of the lamina is acute, the base attenuate and forming a canaliculate petiole that is strongly decurrent down the full length of the internode. The tendrils are up to 34 cm long and mostly coiled. All parts of the foliage may be yellowish green.

*Leaves of the climbing* stem form a broad-winged petiole that is also strongly decurrent. In all other respects, they are consistent with the leaves of the basal stem.

*Lower pitchers* are up to 35 cm tall and 6 cm wide, though usually smaller. The bottom quarter to third of the pitcher is variably inflated, sometimes to the extent that the basal part is bulbous. Above the broad lower section, the pitcher narrows, becoming cylindrical or very slightly infundibular towards the pitcher opening. Wings are usually reduced to ribs, although occasionally, some fringes are present below the pitcher opening. The pitcher opening is oblique and up to 5 cm wide. The peristome is flattened, up to 2.5 cm wide and particularly broad around the sides and below the lid. The peristome is lined with ribs up to 2 mm high, spaced up to 2 mm apart. The ribs are elongated on the inner edge of the peristome to form narrow teeth up to 3 mm long. The lid is ovate, up to 7 cm long and 6 cm wide, with glands densely and evenly distributed over the lower surface. A prominent, triangular, hooked and keeled appendage up to 5 mm long is present on the underside of the lid. The spur is unbranched and up to 5 mm long. The exterior of the lower pitchers is greenish or slightly orange, with narrow orange-red blotches. The interior of the pitcher is white or light yellowish green, lined with small, angular blotches of purple-red. The peristome is red, variably striped with bands of yellow, orange and purple. The lower and upper surface of the lid is bright red.

*Upper pitchers* are up to 40 cm long and 5 cm wide, though usually smaller. The bottom fifth to quarter of the trap is inflated and bulbous, narrowing above this part before becoming cylindrical towards the pitcher opening. Some of the upper pitchers are narrower above the bottom fifth to quarter of the trap, while others are almost cylindrical with almost no narrowing. Wings are indiscernible. The peristome is up to 1 cm wide, cylindrical or slightly flattened, lined with ribs up to 1 mm high, spaced up to 1 mm apart. In all other aspects, including coloration and color variability, the upper pitchers are consistent with the lower ones, although some upper pitchers are entirely green with no blotches.

The *inflorescence* is a panicle, up to 29 cm long, composed of a 19.5 cm-long scape and an additional 9.5 cm rachis bearing pedicels evenly scattered along its length. The pedicels are about 2 cm long, branched and one- or two-flowered. Bracts are absent.

*Indumentum* is present across the foliage, inflorescence and pitchers, consisting of orangey brown hairs up to 1 mm long. 

**Figure 1:**
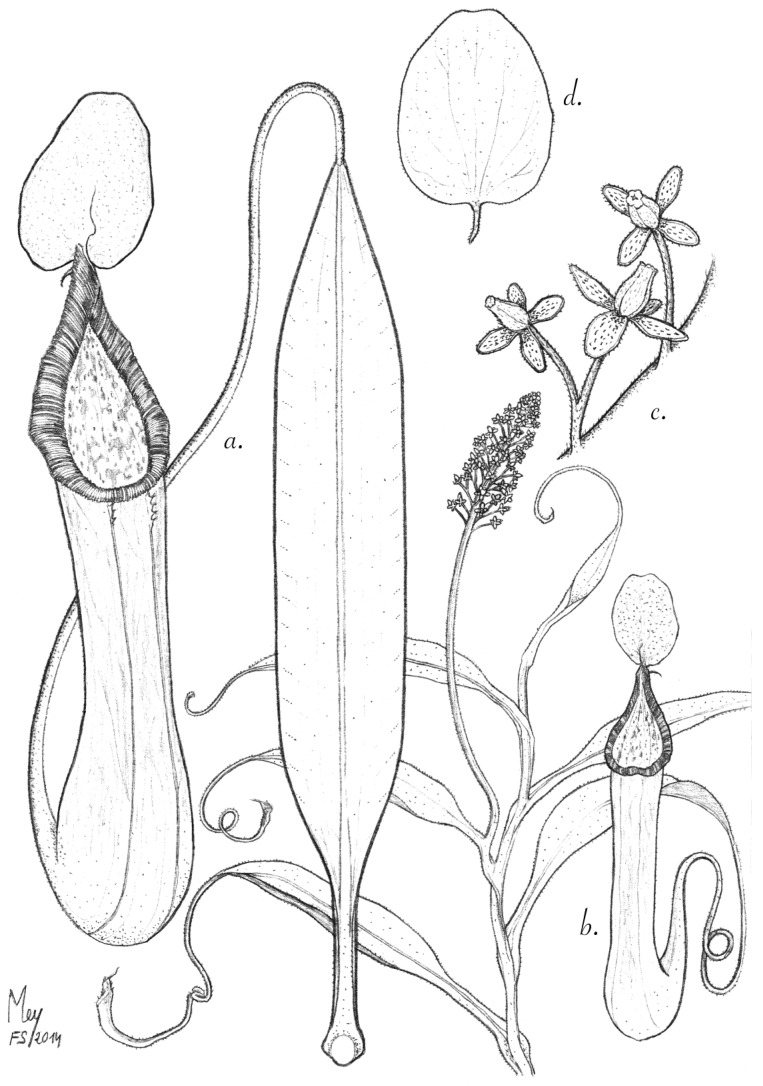
*N. pantaronenesis*. (**a**)Upper pitcher with leaf; (**b**) habit with female inflorescence and upper pitcher; (**c**) partial inflorescence with female flowers; (**d**) upper surface of lid and spur.

*N. pantaronensis* (Figure 1; Appendix Figure A1) is closely related to *N. pulchra* [14] and *N. petiolata* [15]. The most important morphological and ecological differences between these three species are summarized in Table 1. Following the interpretation of Jebb and Cheek [5], these three species belong to the *Reginae* group of species of Danser [15] and represent a group of species on their own among the Philippine species. *N. petiolata* is only known from the Diwata Mountains (including the type locality, Mt. Masay) in the northeast of Mindanao and grows mainly epiphytically. *N. pulchra* represents the most western species and is currently known only from Mt. Kiamo in the Tago range, where it grows terrestrially on ultramafic soils. *N. pantaronensis* is located in between these two species (although geographically closer to *N. pulchra*) and represents a true intermediate between these two; it grows both epiphytically and terrestrially (although not on ultramafic soils, but in an “epiphyte like” mossy substrate) and has both canaliculate and winged petioles, the former on the basal stem and the latter on the climbing stem. 

**Table 1:** The main morphological differences between *N. pantaronensis*, *N. pulchra* and *N. petiolata* (data from [14,15]).

	*N. pantaronensis*	*N. pulchra*	*N. petiolata*
**Lower Pitchers**			
Overall form	Cylindrical, bottom quarter to third variably inflated	Cylindrical, bottom quarter to third variably inflated, often bulbous	Cylindrical, bottom third ovate and variably inflated
Dimension	Up to 35 cm tall and 6 cm wide, though usually smaller	Up to 35 cm tall and 6 cm wide, though usually smaller	Usually up to 21 cm tall and 4.5 cm wide
Wings	Seldom fringed, mostly only ribs	Fringed wings	Fringed wings
Peristome	Flattened, up to 2.5 cm wide	Flattened, up to 2.5 cm wide	Loosely cylindrical
Appendage	Very prominent, triangular, hooked appendage	Rounded, triangular appendage; sometimes only keel present	Only keel; no appendage
**Upper Pitchers**			
Overall form	Bottom fifth to quarter inflated and bulbous, narrowing above this part before becoming cylindrical towards the pitcher opening	Bottom fifth to quarter inflated and bulbous or near spherical, and slightly infundibular above towards the pitcher opening	Cylindrical, bottom fifth to quarter ovate and slightly swollen
Dimension	Up to 40 cm long and 5 cm wide, usually smaller	Up to 34 cm long and 7 cm wide, occasionally up to 42 cm long	Up to 18 cm long and 5 cm wide, occasionally up to 25 cm long
Wings	Indiscernible	Hardly discernible; reduced to ribs if present	Hardly discernible; reduced to ribs if present
Peristome	Cylindrical or slightly flattened, 1–2 cm wide	Flattened, 3 cm wide	Loosely cylindrical, 1–2 cm wide
Appendage	Not hooked, sometimes reduced to a keel	Hooked and rounded, up to 5 mm long near base of the lid	No appendage
**Leaf**			
	Petiole canaliculate on the nonclimbing stem and broad-winged on the climbing stem. Decurrent down full length of internode. Leaves with two longitudinal nerves.	Petiole broadly winged, decurrent down full length of internode, often part way down previous internode as well. Leaves with 3–4 longitudinal nerves.	Petiole canaliculate or winged, clasping the stem. Leaves with four longitudinal nerves.
**Habitat and Distribution**			
	Epiphytic on high trees in lower montane forest at the type locality. Pantaron range and adjacent mountains.	Strictly terrestrial in clearings and montane scrub. Currently only known from Mt. Kiamo.	Epiphytic or rarely terrestrial in lower and upper montane forest. Diwata mountains.

### 2.2. Taxonomic Description of Nepenthes cornuta

*Nepenthes cornuta* Marwinski, Coritico, Wistuba, Micheler, Gronem., Gieray, V.B.Amoroso, spec. nov. 

#### Diagnosis:

Differs from *N. copelandii* Macfarlane in having a narrower lamina, upper pitchers with a distinctive, swollen base and an almost completely cylindrical upper two-thirds (*N. copelandii*: upper pitchers strongly infundibular with a narrow base and proportionally a much wider opening) and noticeably smaller lower pitchers lacking wings (*N. copelandii*: wings always present on lower pitchers).

#### Type:

Philippines, Mindanao Island, Bukidnon Province, Pantaron mountain range, trail from Sitio Mahayag (Barangay St. Peter, Malaybalay City) to Sitio Balaudo, 15.08.2012, *T. Gronemeyer and F. Coritico*, holotype CMUH00008547, Central Mindanao University Herbarium (CMUH), Musuan, Bukidnon, Philippines. 

#### Etymology:

The specific epithet, *cornuta* (lat. cornu = horn), refers to the plant’s horn-shaped upper pitchers.

#### Description:

The *stem* is up to 3 m long and 7 to 8 mm in diameter; the internodes are 4 to 5 cm long.

*Leaves* of the climbing stem are petiolate and linear to lanceolate, up to 30 cm long and 4.5 cm wide. The apex of the leaf blade is acute, the leaf base broadly attenuate and petiolate. The petiole is up to 4 cm long, canaliculate and amplexicaul, clasping between one third and half of the stem. Three parallel longitudinal nerves run on each side of the midrib, on the outer half of the lamina. The tendrils can reach up to 24 cm in length. The foliage and the midrib are green or yellowish green, while the stem and the tendrils can take on a slight red hue when growing in full sunlight.

*Lower pitchers* are up to 15 cm long and 3.4 cm wide. The pitcher opening is ovate and up to 2.8 cm in diameter. The pitcher’s bottom third is bulbous; above this part, the pitchers narrow, sometimes forming a faint hip and becoming cylindrical to slightly infundibular towards their opening. The lid is up to 3.2 cm long, 2.8 cm wide and ovate. The appendage is reduced to a rudimentary keel of up to 1 mm. The spur is up to 3 mm long. The gland distribution is unknown. The peristome has a cylindrical cross-section of up to 5 mm in diameter, positioned at an angle and is lined with very fine ribs. The wings of the pitchers are reduced to ridges, usually without fringes. Occasionally, a few fringes can appear on the top end of those ridges, just below the peristome. The pitchers are green in their bottom third, sometimes taking on a yellowish hue towards the pitcher opening, whilst the upper two-thirds of the pitchers are mottled with red or maroon blotches. The peristome is yellowish to orange or reddish with fine red stripes. The lid is red to maroon with yellowish blotches.

*Upper pitchers* are up to 20 cm long and 4.5 cm wide, the pitcher opening being the widest part. The bottom third is swollen to varying degrees, sometimes displaying a faint hip, and tapers towards the tendril, giving the pitcher its typical, horn-shaped appearance. Above this part, the pitcher narrows faintly and becomes almost completely cylindrical or slightly infundibular towards its opening. The narrowing between the swollen base and the upper part of the pitcher is less pronounced than in the lower pitchers. The lid is up to 5 cm in diameter, ovate to cordate and lacks an appendage. The glands are evenly distributed across the lower surface of the lid. The spur is up to 8 mm long. The peristome is oblique and has a cylindrical cross-section of 5 to 7 mm in diameter. The wings are reduced to inconspicuous vestigial ridges. The pitchers are always green or yellow, except for the interior and the bottom side of the lid, which can have red to maroon speckles. The peristome is green or yellow, often with fine red stripes.

The *inflorescence* is a predominantly two-flowered rachis up to 40 cm long sitting on a scape of up to 21 cm. The individual pedicel can measure up to 15 mm. Bracts are absent.

*Indumentum* is absent from stems, leaves, pitchers and flowers, but the developing pitcher buds are covered with minute, orangey brown hairs.

**Figure 2:**
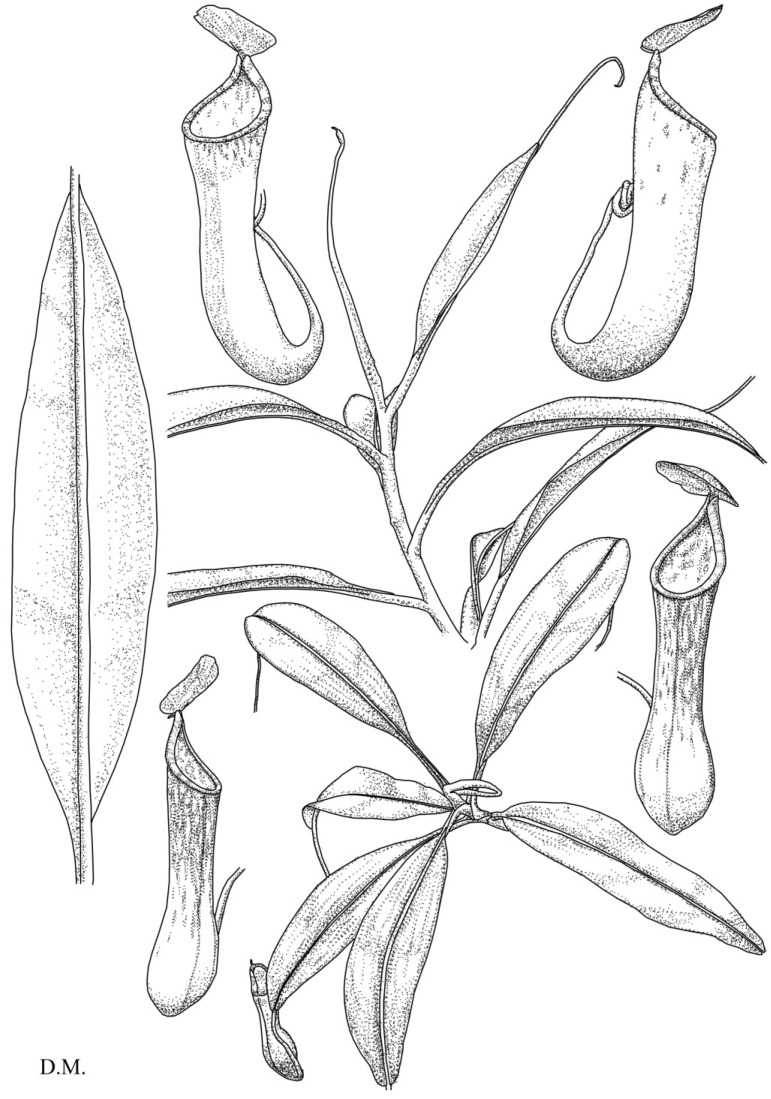
*N. cornuta.* Habit, showing the stem with upper pitchers (**top**). Top view of the rosette with lower pitchers (**bottom**).

*N. cornuta* (Figure 2, Appendix Figure A2) belongs to the *N. alata* complex of species that was recently installed by Jebb and Cheek [5,8]. Together with *N. copelandii* [16] and *N. ceciliae* [13], it forms a subgroup with the upper pitchers, which are not widest in their basal half [5]. The morphological differences between these species are listed in Table 2. 

**Table 2:** The main morphological differences between *N. cornuta*, *N. copelandii* and *N. ceciliae* (data from [13,16]).

	*N.* *cornuta*	*N.* *copelandii*	*N.* *ceciliae*	
**Lower Pitchers**				
Overall form	Elongate, with a bulbous bottom third, narrowing above this part, sometimes with a faint hip, cylindrical to slightly infundibular towards the pitcher opening	Elongate, almost cylindrical, narrowing only faintly above the bottom third of the pitcher	Squat, with a bulbous bottom half, the upper half widening slightly towards the opening	
Dimensions	Up to 15 cm long and 3.4 cm wide in the lower part	Up to 26 cm long and 5.5 cm wide at the peristome	Up to 10 cm long and 3 cm wide	
Wings	Reduced to ridges	Present, up to 1.4 cm wide, fringes up to 12 mm long	Present, up to 4 mm wide, fringes up to 3 mm long	
Peristome	Cylindrical, up to 5 mm wide	Loosely cylindrical, up to 1.2 cm wide	Flattened, up to 4 mm wide	
Appendage	Reduced to a rudimentary keel	Absent or reduced to a rudimentary keel	Present, 2 mm long	
**Upper Pitchers**				
Overall form	Elongate, bottom third variably swollen, faint narrowing towards the middle, cylindrical to slightly infundibular in the upper half, the peristome being the widest part	Entirely infundibular, the peristome being the widest part, no narrowing in the middle	Infundibular bottom third, cylindrical or slightly infundibular upper part, the peristome being the widest part, no narrowing in the middle	
Dimensions	Up to 20 cm long and 4.5 cm wide at the peristome	Up to 12 cm long and 4.5 cm wide at the peristome	Up to 10 cm long and 4 cm wide at the peristome	
Wings	Reduced to faint ridges	Absent	Reduced to faint ridges	
Appendage	Absent	Absent or reduced to a rudimentary keel	Present, 2 mm long	
**Habitat and Distribution**				
	Terrestrial on ultramafic soils on clearings or open scrub. Pantaron range.	Terrestrial or epiphytic. Mt. Apo and Mt. Pasian.	Strictly terrestrial on ultramafic soils. Currently only known from Mt. Kiamo.	

### 2.3. Taxonomic Description of Nepenthes talaandig

*Nepenthes talaandig* Gronem., Coritico, Wistuba, Micheler, Marwinski, Gieray, V.B.Amoroso, spec. nov. 

#### Diagnosis:

Differs from *N. cornuta* Marwinski in having bulbous lower pitchers with a flattened, crenellated peristome (*N. cornuta*: slender lower pitchers; cylindrical towards the pitcher opening; with cylindrical peristome) and having a winged petiole that clasps the stem (*N. cornuta*: canaliculate petiole). 

#### Type:

Philippines, Mindanao Island, Bukidnon Province, Pantaron mountain range, trail from Sitio Mahayag (Barangay St. Peter, Malaybalay City) to Sitio Balaudo, 15.08.2012, *T. Gronemeyer and F. Coritico*, holotype CMUH00008624, Central Mindanao University Herbarium (CMUH), Musuan, Bukidnon, Philippines.

#### Etymology:

The specific epithet was chosen to acknowledge the indigenous tribe of the Talaandig. *N. talaandig* occurs on the ancestral territory of the Talaandig communities of east Bukidnon. 

#### Description:

*The stem* is up to 8 m long, cylindrical in the cross-section and 7–8 mm in diameter. The internode length is 3.5–6 cm. 

*Leaves* of the climbing stem are elliptic, up to 35 cm long and 4.5 cm wide, with four equally distributed longitudinal veins on each side of the midrib and numerous pinnate veins running obliquely towards the leaf margins. The apex of the lamina is acute, the base attenuate and forming a narrow-winged petiole 6 cm in length that clasps one third of the stem. Pitchers are on tendrils 16–20 cm long. 

*Lower pitchers* are bulbous or ovate, 10 cm tall and 5 cm wide. The pitcher narrows slightly towards the opening. Wings are up to 7 mm wide, fringed with filaments up to 7 mm long and run down the front of the trap. The pitcher opening is at a prominent angle, up to 3.5 cm wide and acuminates towards the lid. The peristome is flattened, up to 1.5 cm wide and crenellated. It extends into a neck towards the lid and is densely lined with ribs approximately 1 mm high. The lid is ovate to cordate, up to 5.5 cm long and 5 cm wide, with glands evenly distributed across the lower surface. A rounded, triangular appendage up to 3 mm long, sitting on a keel, is present on the underside of the lid. The spur is branched and up to 6 mm long. The exterior of the lower pitchers is rusty red to violet, with narrow blotches of reddish purple. The interior of the pitcher is white and sometimes lined with angular blotches of purple. The peristome is dark red and not striped. 

*Rosette pitchers*, which are squatter and considerably smaller than the lower pitchers, have been observed *in situ* on young plants. In all other respects, they resemble the lower pitchers. 

*Upper pitchers* are slender, around 20 cm long and 7 cm wide. The bottom third of the trap is slightly inflated. The pitcher narrows towards a cylindrical middle section before it widens towards the oblique pitcher opening; the latter being 4.5 cm wide. Wings are reduced to ribs throughout. The peristome is not crenellated, is cylindrical to flattened, up to 1.2 cm wide and extends into a short neck. The color of the peristome is yellow with discrete stripes of red. The lid is ovate to orbicular with a diameter of 4 cm. A prominent triangular appendage of 2.5 mm on a keel is present; the spur is 6 mm long and branched. The upper pitchers are colored yellowish-green, often with red speckles or blotches. The pitcher interior is cream-colored without blotches. 

The *inflorescence* is a panicle, composed of a rachis up to 40 cm long sitting on a 20-cm scape. It bears branched two-flowered partial peduncles 5 mm long. Bracts are present. 

*Indumentum* is sparse, if present at all. 

**Figure 3:**
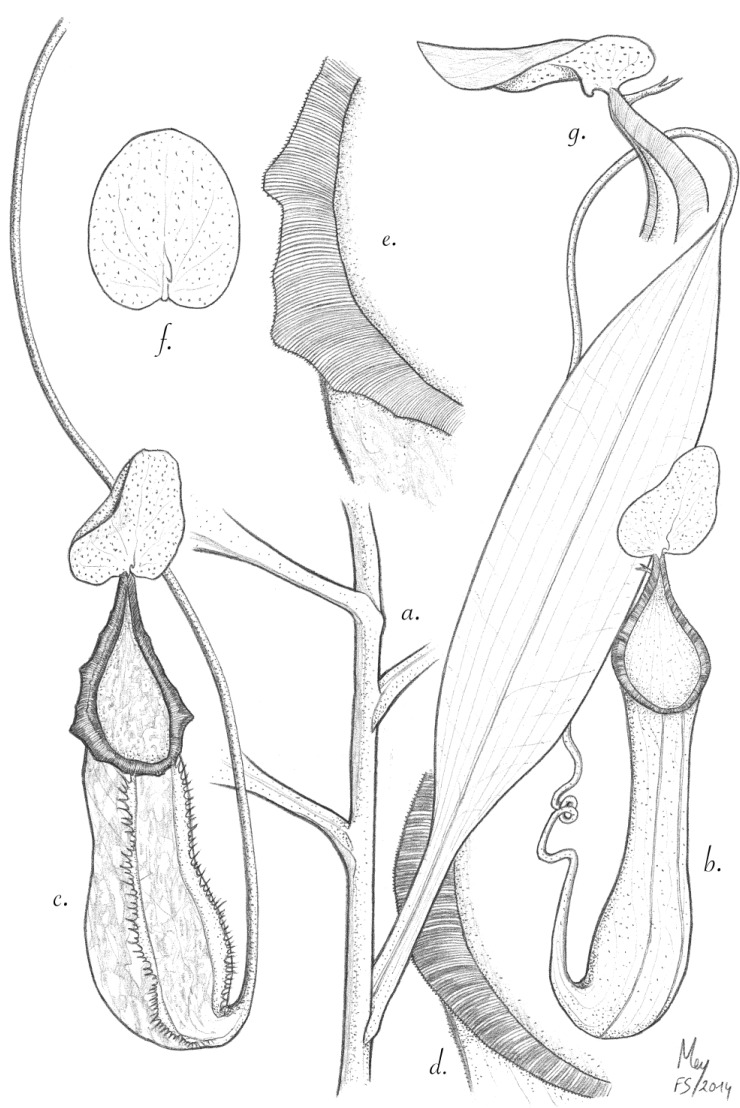
*N. talaandig*. (**a**) Climbing stem with leaf; (**b**) upper pitcher; (**c**) lower pitcher. (**d**) Detail of the upper pitcher peristome; (**e**) detail of the lower pitcher peristome; (**f**) the underside of the lid; (**g**) appendage and spur.

*N. talaandig* (Figure 3, Appendix Figure A3) is not directly related to any other Philippine species, due to its unique ovate, bulbous lower pitchers with a prominently angled mouth that acuminates towards the lid. The lower pitchers bear superficial similarities with those of some Sumatran species, such as *N. longifolia* Nerz or *N. bongso* Korth., whereas the upper traps share similarities with those of species of the *N. alata* group (usually ovoid-cylindrical upper pitchers with an appendage on the basal part of the lower surface) [5].

### 2.4. Taxonomic Description of Nepenthes amabilis

*Nepenthes amabilis* Wistuba, Gronem., Micheler, Marwinski, Gieray, Coritico, V.B.Amoroso, spec. nov. 

#### Diagnosis:

Differs from *N. pantaronensis* Gieray in having spathulate-ovate, approximately 10 cm long and 3.5 cm wide leaves with an obtuse apex (*N. pantaronensis:* up to 28 cm long and narrowly acute apex) and having mostly cylindrical to slightly infundibular upper pitchers that are only 10–15 cm tall and distinctly contracted in the region below the peristome (*N. pantaronensis:* clearly inflated in the lowest quarter and up to 40 cm tall). 

#### Type:

Philippines, Mindanao, Mt. Sumagaya, trail from Barangay Mat-I (Municipality of Claveria) to the summit 19.08.2012, *T. Gronemeyer and A. Wistuba*, holotype CMUH00008635 (male flower), isotype CMUH00008637 (female flower), Central Mindanao University Herbarium (CMUH), Musuan, Bukidnon, Philippines.

#### Etymology:

The specific epithet, *amabilis* (lat. amabilis = lovely), refers to the extraordinary beauty of the compact specimens with very colorful pitchers and mostly striped peristomes that were observed *in situ*. 

#### Description:

The *stems* are often very short. Plants start to grow upright at a young age and are supported by surrounding vegetation, mostly low shrubs. The stem of short plants is green and often almost without any significant internodes. Occasionally, in more shady locations and where the stems can scramble along other plants as support, plants develop climbing stems that reach 2–3 m in length. These vines are almost round in the cross-section, 8 mm in diameter, green to dark red and with internodes that are 10–15 cm long.

*Leaves* of the vines and short stems are broadly spathulate-ovate, 10 cm long and 3.5 cm wide, with a broad, winged and 1 cm-wide petiole. The leaves are green with yellowish to reddish midribs. The leaf attachment is decurrent, running 1–3 cm down the stem. One to two longitudinal nerves run parallel to the midrib in the outer half of the lamina. Pitchers are attached to tendrils up to 13 cm long.

*Lower pitchers* of rosette plants are unknown; plants with lower pitchers could not be identified with certainty at the type locality. 

*Upper pitchers* of short and climbing stems are 10–15 cm high, approximately 3 cm wide, mostly cylindrical, slightly infundibular in the lower third and distinctly contracted in the region below the peristome. The bottom fifth is often slightly ventricose with a slight waist above. The pitcher wings are reduced to ribs. Only occasionally in some pitchers, approximately 5 mm-wide wings bearing two or three fringes measuring approximately 5 mm in length are visible close to the peristome. The peristome is cylindrical and approximately 4 mm wide. The spur on the pitcher lid is unbranched, filiform and 3 mm long. The pitcher mouth is round, slightly elevated at the front and elongated towards the back with a slightly developed neck. The lids are 3 cm long, 3.5 cm wide, orbicular, sometimes with a small appendage near the base of the lid, which is mostly reduced to a keel. The glands are evenly distributed across the lower surface of the lid. The exterior of the upper pitchers is yellowish green to orange, unevenly suffused with red or with red blotches. The interior of the pitcher is whitish with numerous purple blotches that often shine through the pitcher wall. The peristome is yellowish green with numerous red stripes. The lid is yellowish with numerous red blotches.

The *inflorescence* is a panicle composed of a 15 cm-long scape and an additional 15 cm rachis bearing two-flowered, 20 mm-long pedicels. The anther column is up to 14 mm and the ovary 5 mm long, containing ripe seeds; the tepals are up to 7 mm long and ovate. 

A prominent *indumentum* is present across the foliage, the inflorescence, especially the margins of young leaves, and pitchers, consisting of brownish hair up to 1 mm long.

**Figure 4:**
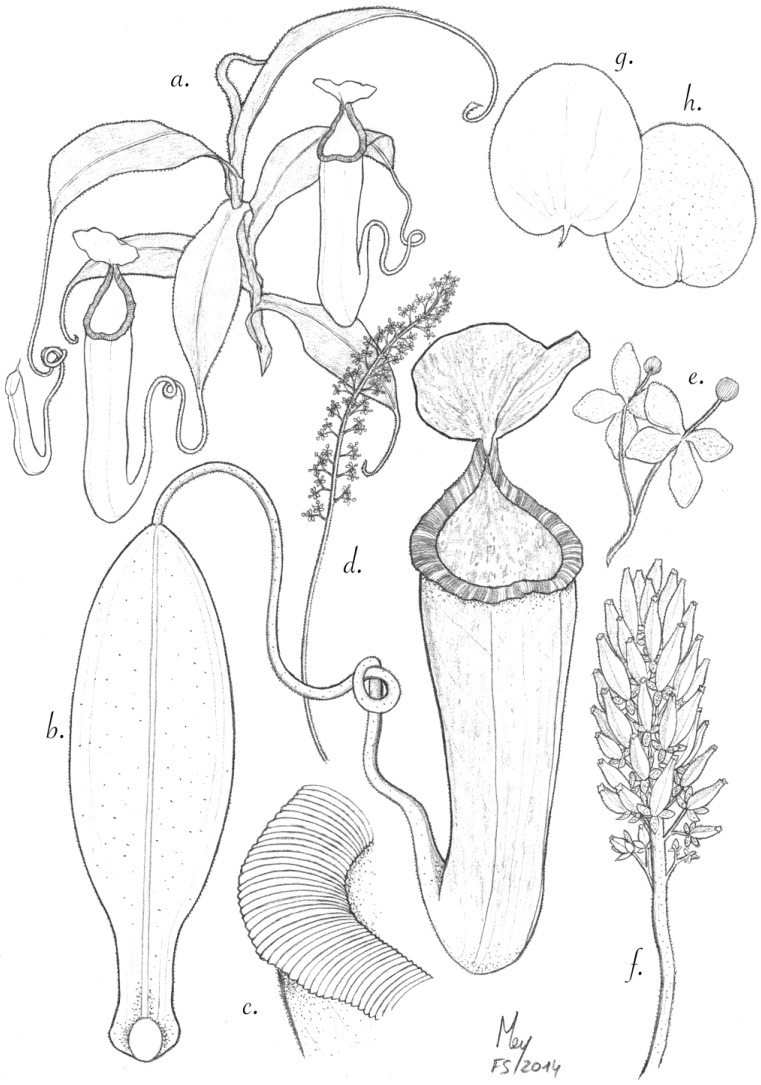
*N. amabilis*. (**a**) Habit with upper pitchers; (**b**) leaf with upper pitcher; (**c**) detail of peristome; (**d**) male inflorescence; (**e**) partial inflorescence with male flowers; (**f**) infructescence; (**g**) the upper surface of lid with spur; (**h**) the underside of the lid. Rosette plants with lower pitchers could not be identified with certainty at the type locality and are thus not depicted.

*N. amabilis* (Figure 4 and Appendix Figure A4) is apparently not related to *N. pantaronensis*, with which it grows sympatrically on Mt. Sumagaya, nor to any other species occurring on Mindanao. Thus, it is unlikely that this species could be confused with another in the field.

### 2.5. Habitat

*N. cornuta* and *N. talaandig* occur in the Pantaron mountain range at altitudes around 1,000 m. Most observed populations occur terrestrially on ultramafic soil in clearings and on bright ridges in lower montane forest. 

At the type locality, *N. pantaronensis* grows at intermediate altitudes as a strict epiphyte on moss-laden branches of large trees in lower montane forest. 

On Mt. Sumagaya, smaller, stunted stands of *N. pantaronensis* occur terrestrially in lower and upper montane forest in mossy and open substrate. 

The three species grow in the Pantaron range sympatrically with *N. surigaoensis* and *N. truncata.* Although no natural hybrids with any of these species have been observed *in situ*, hybridization events are likely. 

*N. amabilis* grows exclusively in open areas of grass and shrub vegetation on Mt. Sumagaya from 1,600 m up to the summit, which stands at 2,247 m. It is completely absent from the lower and upper montane forest that covers the slopes of this mountain. Plants of possible hybrid origin with *N. pantaronensis* have been observed *in situ*. 

### 2.6. Infauna

At the type locality of *N. cornuta*, several ant colonies (*Crematogaster* sp.) were found in dead or dying pitchers of this species. The ants obstruct the pitcher opening with a greyish matter, probably made out of vegetal detritus and dirt. This ‘lid’ has small entry holes along the pitcher walls. The bottom of the pitchers is pierced with one or two round holes, draining them of their digestive fluid. These holes also serve as “emergency exits” for the ants when disturbed. The presence of those insects on some pierced functional pitchers without a nest might suggest that the ants first pierce and drain functional pitchers before colonizing them. A similar colonization has been observed before in other *Nepenthes* species, e.g., *N. macfarlanei* Hemsl*.* in Malaysia [17] and *N.* *maxima* Reinw. on the lower slopes of the Maoke Mountains in West Papua (personal observation by the authors).

### 2.7. Conservation Status

The Pantaron range is currently visited only by the indigenous people of the Talaandig tribe, who cross the mountains to reach remote, temporarily inhabited villages or with the purpose of hunting and collecting rattan or resin. The impact of poaching on the population of *Nepenthes* spp. is low. 

However, the foothills of the Pantaron range are completely deforested for agriculture, and illegal logging may take place in the forests. Therefore, habitat destruction has the biggest impact on the population of the *Nepenthes* spp. in this region.

It may be anticipated that both *N. talaandig* and *N. cornuta* are widely distributed in the Pantaron range, as they do not occur on isolated peaks. Since only few data have been recorded about the distribution of *N. talaandig* and *N. cornuta*, these two taxa are assessed here as VU (vulnerable) according to the IUCN Red List criteria [18]. 

*N. pantaronensis* is known both from the Pantaron range and Mt. Sumagaya, and thus, a wider distribution is documented. It is assessed here as NT (near threatened). 

*N. amabilis* is currently only known from the upper slopes of Mt. Sumagaya. It has not been recorded in the explored areas of the Pantaron range, nor on Mt. Kiamo in the Tago range. Mt. Sumagaya is only visited by the indigenous people of the Higaonon tribe, who cross the mountains to hunt deer, and by dispersed groups of the NPA who hide in the mountains. The impact of poaching is low. However, as no comprehensive data about the distribution of this taxon have been recorded, it is classified here as EN (endangered). 

## 3. Experimental Section

Field research was carried out during August, 2012. All data and dimensions were either collected in the field from live plants or from the respective herbarium types cited above.

The Pantaron range in the area of Barangay St. Peter (Malaybalay City) was explored after an access permit was granted by the Bukidnon Resource Management Foundation and the Biodiversity Management Bureau (BMB) of the Department of Environment and Natural Resources (DENR), and with the consent of the local indigenous people of the Talaandig tribe. 

Mount Sumagaya was explored with the consent of the local military authorities in Barangay Mat-I (Municipality of Claveria, Misamis Oriental Province, Philippines) and the local indigenous people of the Higaonon tribe.

Herbarium specimens were collected under a collection permit granted to VB (Permit No. MOA 10-2-5 from 2012). 

Cropped plant material was placed between paper sheets *in situ* and processed subsequently using standard methods at the herbarium of the Central Mindanao University.

Photographs were made from suitable, representative plant specimens *in situ*.

Image credits: Botanical drawings in Figure 1, Figure 3 and Figure 4: FSM. Botanical drawing in Figure 2: DM. Photos in Appendix Figures A1–4: AW.

## 4. Conclusions

Carnivorous pitcher plants are widespread within the Malesian biogeographical region comprising the Malayan peninsula and the islands of Sumatra, Borneo, Sulawesi and the Philippines. Especially in the Philippines, the majority of species are known from centers of diversity and endemism. Several species are restricted to one single mountain top or a mountain ridge. 

A field research trip to the formerly unexplored central cordillera of Mindanao led to the discovery of four new taxa of carnivorous pitcher plants, namely *N. amabilis*, *N. cornuta*, *N. pantaronensis* and *N. talaandig*. 

While *N. cornuta* and possibly *N. talaandig* belong to the *N. alata* group of species, *N. pantaronensis* is clearly related to *N. pulchra* and *N. petiolata* and, thus, belongs to the *Reginae* group.

*N. amabilis* stands clearly on its own among the Philippine *Nepenthes* species. 

Other species in the central cordillera include *N. ceciliae*, *N. pulchra*, *N. surigaoensis* and *N. truncata*. However, the latter two species are widespread and also occur in other regions of Mindanao.

As with many other species of *Nepenthes* in the Philippines, the future of all species of the central cordillera is highly dependent on habitat conservation. Mt. Kiamo is not currently a protected area and, thus, the conservation status of the two endemic species, *N. ceciliae* and *N. pulchra*, needs to be monitored closely in the future. 

If the current status “key biodiversity area” for the Pantaron range and the Kimangkil massif, including Mt. Sumagaya, leads to permanent protection combined with careful monitoring of the relevant protective measures, at least the four endemic *Nepenthes* species we described in this article would be assured preservation in the long term. 

## Figures and Tables

**Figure A1:**
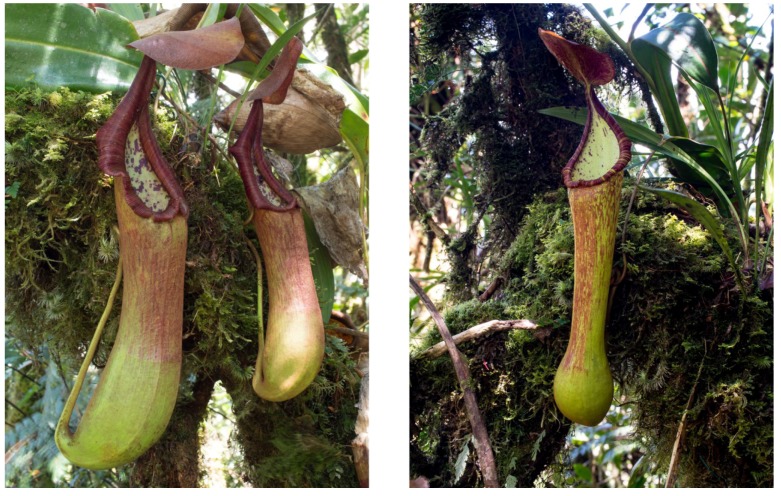
*N. pantaronensis*: photos of a lower (**left**) and upper pitcher (**right**) taken at the type locality.

**Figure A2:**
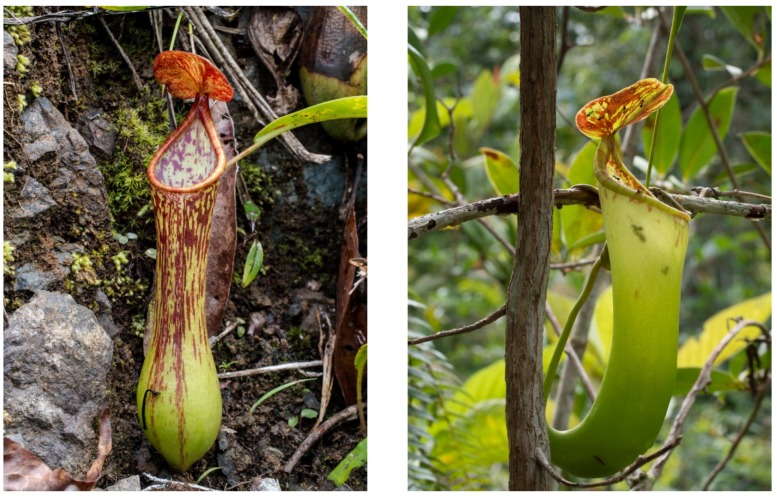
*N. cornuta*: photos of a lower (**left**) and upper pitcher (**right**) (with *Crematogaster* sp.) taken at the type locality.

**Figure A3:**
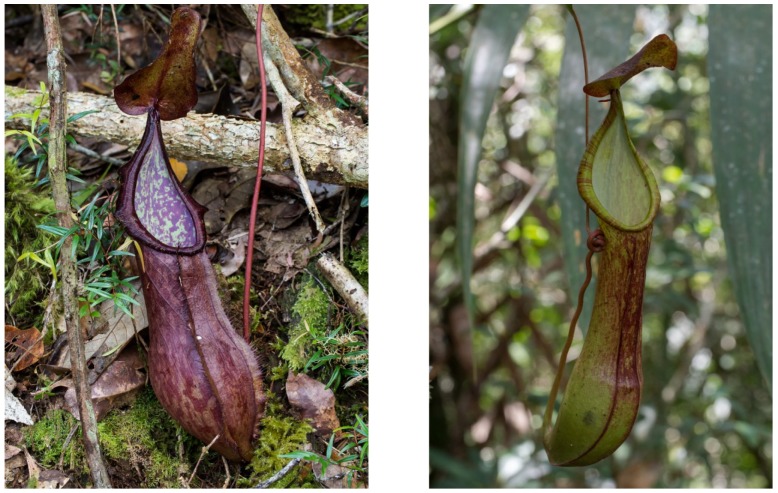
*N. talaandig*: Photos of a lower (**left**) and upper pitcher (**right**) taken at the type locality.

**Figure A4:**
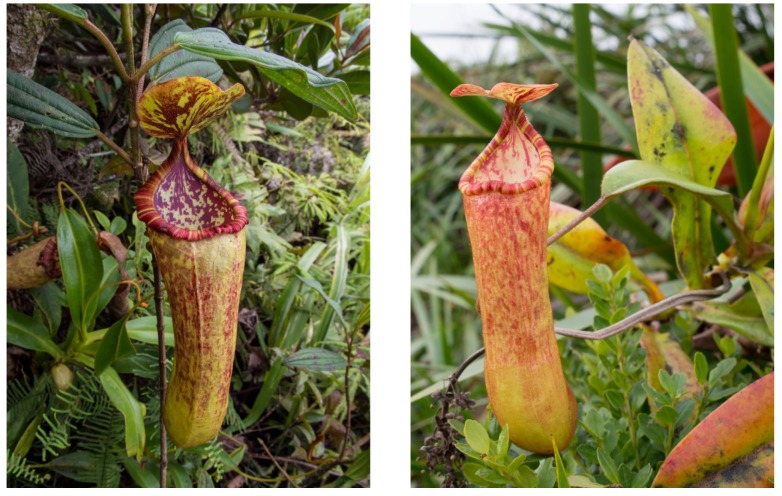
*N. amabilis*: photos of two pitchers taken at the type locality. In the **left** panel, the distinct leaf shape can be seen.
